# Dynamic ROS Production and Gene Expression of Heifers Blood Neutrophil in a Oligofructose Overload Model

**DOI:** 10.3389/fvets.2020.00211

**Published:** 2020-04-21

**Authors:** Shuaichen Li, Jiafeng Ding, Lihong Jiang, Muhammad Abid Hayat, Qiaozhi Song, Yuepeng Li, Xianhao Zhang, Jiantao Zhang

**Affiliations:** ^1^College of Veterinary Medicine, Northeast Agricultural University, Harbin, China; ^2^Heilongjiang Key Laboratory for Laboratory Animals and Comparative Medicine, Harbin, China

**Keywords:** cow, oligofructose overload, neutrophil, reactive oxygen species, gene expression

## Abstract

Alimentary oligofructose (OF) overload can induce several diseases in cattle, such as ruminal acidosis, laminitis, and synovitis. The role of blood polymorphonuclear neutrophil (PMN) remains unclear during OF overload. The aim of this study was to investigate the dynamic changes in reactive oxygen species (ROS) production and the expression profile of genes in blood PMN in a model of OF overload. Twelve clinically healthy and non-pregnant Chinese Holstein heifers, aged between 18 and 26 mo, weighing 335–403 kg, BCS (5-point scale) ranges 2.7–3.3 were used for the experiments. OF heifers (*n* = 6) received 17 g/kg of BW oligofructose dissolved in 2 L/100 kg of BW tap water and the CON heifers (*n* = 6) received 2 L/100 kg of BW tap water. Blood PMN was isolated for each heifer 0, 6, 12, 18, 24, 36, 48, 60, and 72 h after administration. PMN was analyzed either by endogenous and phorbol myristate acetate (PMA)-induced ROS production or by quantitative real-time PCR. After 12 h, PMA-induced ROS production decreased, which was sustained until 48 h. The expressions of inflammation markers (IL1α, IL1β, IL6, IL10, TNFα, STAT3, TLR4, MMP9, and HP) and eicosanoids (ALOX5, ALOX5AP, and PLA2G4A) were upregulated. The expression of adhesion and migration (CXCR2, CXCL8, CD62L, ITGA4, ITGAM, and ITGB2) in OF heifers was increased compared with CON heifers. The expression of oxidative stress (SOD2 and S100A8) was upregulated, while SOD1 and MPO were downregulated. In metabolism and receptor genes, the expressions of GRα and INSR decreased after 12 h, while Fas increased until 6 h and then decreased at 18 h. The expression of LDHA and PANX1 did not show any differences after OF overload. These findings indicate that OF overload induced systemic activation of PMN, which provides a step toward a better understanding of the role of innate immune responses in response to oral OF administration.

## Introduction

Oligofructose (OF) overload in cattle has been widely studied in acute ruminal acidosis (ARA), laminitis, and synovitis (1–3). Excessive intake of fermentable carbohydrates leads to a change in microbiota and fermentation rate. The concentrations of lactate was increased in the rumen fluid and absorbed into the systemic circulation in acute rumen acidosis (4). In addition to lactate, lipopolysaccharide (LPS) has also been considered to be a major factor in the development of ruminal acidosis. LPS is generally assumed to be translocated into the blood stream during experimentally induced subacute ruminal acidosis (SARA) (5–7). These substances cause systemic inflammatory responses, but dynamic changes of innate immunity still remain unclear in ARA, particularly the role of polymorphonuclear neutrophil (PMN).

PMN is also called granulocytes including neutrophils, eosinophils, and basophils. PMN is the first line of cellular defense in systemic inflammation, including neutrophil migration and activation. It has been demonstrated a decline in platelet activating factor (PAF)-induced reactive oxygen species (ROS) production and L-selectin shedding in heifers with acute ruminal acidosis induced by OF overload (2). Additionally, cows fed with higher-energy diets had altered the expressions of multiple genes of blood PMN compared with cows that received control diets (8). Furthermore, *in vitro* experiments showed that D-lactic acid has been reported to improve neutrophil adhesion and elevate the neutrophil extracellular trap release (9). This study investigated the dynamic changes of ROS production and the expression of selected genes (Table 1) within 72 h of the course of OF overload. We hypothesized OF treatment would alter superoxide anion generation, and target transcript expression across inflammatory response, adhesion, migration, oxidative stress, synthesis of eicosanoids, and receptors that orchestrate metabolic functions in the circulating pool of neutrophils.

**Table 1:** Gene symbol, Gene name, and PCR efficiency used to analyze gene expression by quantitative reverse transcription PCR.

**Gene symbol**	**Gene name**	**PCR efficiency (%)**
**Inflammation**		
*HP*	Haptoglobin	90
*IL1α*	Interleukin 1 α	110
*IL1β*	Interleukin 1 β	86
*IL10*	Interleukin 10	89
*IL6*	Interleukin 6	105
*MMP9*	Matrix metallopeptidase 9	99
*STAT3*	Signal transducer and activator of transcription 3	91
*TLR4*	Toll like receptor 4	100
*TNFα*	Tumor necrosis factor α	85
**Adhesion/Migration**		
*CD62L*	L selectin	92
*CXCL8*	Chemokine (C-X-C motif) ligand 8	98
*CXCR2*	Chemokine (C-X-C motif) receptor 2	85
*ITGA4*	Integrin subunit alpha 4	76
*ITGAM*	Integrin subunit alpha M	84
*ITGB2*	Integrin subunit beta 2	102
**Oxidative stress**		
*MPO*	Myeloperoxidase	94
*S100A8*	S100 calcium binding protein A9	87
*SOD1*	Superoxide dismutase 1	88
*SOD2*	Superoxide dismutase 2	82
**Eicosanoids**		
*ALOX5*	Arachidonate 5-lipoxygenase	89
*ALOX5AP*	Arachidonate 5-lipoxygenase-activating protein	87
*PLA2G4A*	Phospholipase A2 group IVA	87
**Metabolism/Receptor**		
*Fas*	TNF receptor superfamily member 6	104
*GRα*	Glucocorticoid receptors α	92
*INSR*	Insulin receptor	98
*LDHA*	lactate dehydrogenase A	85
*PANX1*	Pannexin 1	89
**Control genes**		
*ACTB*	β-actin	97
*UXT*	Ubiquitously expressed prefoldin like chaperone	101
*GAPDH*	Glyceraldehyde-3-phosphate dehydrogenase	91

## Materials and Methods

### Ethics Statement

All procedures, treatments, and animal care were conducted under the approval of Institutional Animal Care and Use Committee of Northeast Agricultural University (approved protocol number SRM-13) in accordance with Laboratory animal-Guideline for ethical review of animal welfare.

### Animals

Twelve clinically healthy non-pregnant Chinese Holstein heifers, with normal locomotion, and without serious claw lesions, aged between 18 and 26 mo (20.67± 3.01 mo), weighing between 335 and 403 kg (379.71 ± 19.87 kg), and BCS (5-point scale) between 2.7 and 3.3 (10) were selected and randomly divided into two groups: OF and control (**CON**). These heifers were permitted to acclimatize for 30 days and were housed in tie stalls with concrete floor throughout the experiment. All heifers had free access to water and fed mixed grass-lucerne hay *ad libitum* during the adaptive and experimental period. The jugular vein was catheterized 3 days before the experiment started.

### Experimental Design and Treatments

The administration of OF was based on the method described by Danscher et al. (1). OF heifers (*n* = 6) received 17 g/kg of BW oligofructose (food grade, Shandong Bailong Group, China) dissolved in 2 L/100 kg of BW tap water at time 0 h. The solution was given via an oral-rumen tube (length 2.2 m, diameter 25 mm). CON heifers (*n* = 6) received 2 L/100 kg of BW tap water. For 3 days before the primary overload, 5% of the primary dose was provided to the OF heifers daily, while water was provided to the CON heifers. All heifers were treated from 06:00 a.m. in blocks of 4 starting on 3 consecutive days. Considering animal welfare, supportive therapy was provided in the form of calcium borogluconate at 18 h, and Ringer's solution (Heping Animal Medicine Co., Ltd, Harbin, China) and sodium bicarbonate at 18 and 24 h after OF overload. The sample size rationale was estimated by G^*^Power (ver. 3.1.3) analysis software as previously described (2), which the parameters employed were the effect size *f* = 0.5; α error = 0.05; power (1–α) = 0.8; and number of measurements = 6.

Acute ruminal acidosis is defined as ruminal fluid pH <5.0 along with evident clinical signs (11); Therefore Ruminal fluid was collected by oral-rumen tube prior to and following overload every 12 h, and pH was immediately measured (Benchtop pH meter, Mettler Toledo Inc., Switzerland) to confirm the presence of ruminal acidosis. Ruminal fluid was also used for further research to evaluate the changes of rumen microorganisms and metabolites (unpublished data). Clinical examination were performed on all heifers including eating behavior, feces consistency, heart rate, respiration frequency, and rectal temperature every 6 h.

### Blood Collection

At 0, 6, 12, 18, 24, 36, 48, 60, and 72 h after OF overload, 60 ml of blood was collected into acid citrate dextrose evacuated tubes, which were inverted and pooled by transfer into three conical sterile tubes. Two tubes (20 mL blood each) for RNA extraction were placed on ice and one tube (20 mL blood) for ROS determination was kept at room temperature.

### PMN Isolation

Neutrophils were isolated with modifications according to previously described procedures (8). After blood collection, the following centrifugation process was identical except that the centrifugal temperature was kept at room temperature to assess ROS production. Tubes were centrifuged for 20 min at 1,000 × g at 4°C. The plasma, buffy coat, and up to one-third of red blood cells were removed and discarded. Deionized water (18 mL) was added at 4°C to lyse red blood cells, followed by addition of 2 mL of 10 × PBS at 4°C to restore isotonicity. Samples were then centrifuged at 200 × g for 10 min at 4°C. Subsequently, samples were washed twice with 1 × PBS and re-collected by centrifugation at 850 × g for 5 min at 4°C. TRIzol reagent (Invitrogen, Carlsbad, CA, USA) was added to isolated PMN. After homogenization, the suspension was transferred to DNase-/RNase-free microcentrifuge tubes and stored at −80°C until further analysis.

### Assessment of ROS Production

Trypan blue straining was used to test the viability (>97%) of PMN. The purity (>95%) was assessed in Wright-Giemsa stain by light microscopy. All samples were analyzed in duplicate. PMN (1 × 10^6^ cells/mL) was resuspended in phenol red-free RPMI 1640 medium and stimulated with 20 μM phorbol myristate acetate (PMA, dissolved in DMSO, Beyotime Biotechnology, Nanjing, China) and non-stimulated cells received equal DMSO. Either 20 μM 2′, 7′-dichlorofluorescin diacetate (DCF-DA, dissolved in DMSO, Jiancheng Bioengineering Institute, Nanjing, China) or equal DMSO were added immediately. All samples were incubated for 30 min in 5% CO_2_, at 95% humidity and 37°C. Arbitrary fluorescence of ROS production was measured using the fluorescence spectrophotometer F4500 (Hitachi, Japan).

### RNA Extraction and cDNA Synthesis

Total RNA was extracted from neutrophils using the TRIzol reagent, following the manufacturer's instructions. Briefly, to remove DNA, total RNA was separated with chloroform followed by acid phenol:chloroform. Total RNA was precipitated with isopropanol and then cleaned with 75% ethanol. Both the quantity and purity of total RNA was measured using NanoDrop™ One Microvolume spectrophotometry (Thermo Scientific, USA). The integrity of RNA was determined via 1% agarose gel electrophoresis.

A total of 1 μg RNA per sample was reverse transcribed using the PrimeScript™ RT reagent Kit with gDNA Eraser (Takara, Dalian, China) following the instructions of the manufacturer. Subsequently, cDNA was diluted 1:4 with DNase/RNase free water and stored at −20°C.

### Quantitative Real-Time PCR Analysis

Primers were designed based on previous literature and the primer details are presented in the Supplemental Material. The specificity of the primer sequences was verified by BLAST (http://blast.ncbi.nlm.nih.gov/). Primers were purchased from Sangon Biotech Co., Ltd, Shanghai, China.

Quantitative PCR was conducted in LightCycler 480 (Roche, Germany) following the manufacturer's protocol using SYBR Premix Ex Taq™ II (Takara, Dalian, China). Briefly, 2 μL of cDNA was combined with 10 μL of SYBR green dye, 0.8 μL of each primer solution (forward and reverse, each 10 μM), and 6.4 μL of DNase/RNase free water. The reaction parameter was: 1 cycle of 95°C for 1 min, followed by quantification analysis mode 40 cycles of 95°C for 5 s and 60°C for 1 min, ending with melting curve analysis mode 95°C for 5 s, 60°C for 1 min, increasing 0.5°C until 95°C, followed by 50°C for 30 s.

The cycle threshold (Ct) was calculated with the LightCycler 480 software (version 1.5.0, Roche, Germany). PCR efficiency were determined using serial dilutions of pooled cDNA and the results are presented in Table 1. The Ct values were standardized using the geometric mean of three reference genes (ACTB, GAPDH, and UXT) based on previous research (12, 13) and were calculated based on the ΔΔCt method taking quantitative PCR efficiency into account (14). The fold change was calculated as shown in equation:

(1) $$\begin{array}{l} fold\;change=\frac{{\left(E_{target}\right)}^{\Delta Ct_{target}\left(time\;point\;-time\;0\right)}}{{\left(E_{ref}\right)}^{\Delta Ct_{ref}\left(time\;point\;-time\;0\right)}} \end{array}$$

### Statistical Analysis

Statistical analysis were performed using GraphPad Prism version 7.04 (GraphPad Software Inc., San Diego, CA, USA). Gene expression data were converted to log-2 values prior to statistical analysis. Two-way repeated measures ANOVA was performed to determine the interaction between time and group. The group (G) and time (T) are considered as fixed effect according to the following model:

(2) $$\begin{array}{l} Y=\mu +G+T+G\;*\;T+e \end{array}$$

Where Y is the dependent variable, μ is population mean, and e is the random error. Gene expression data were then analyzed by one-way repeated measures ANOVA within groups to determine whether a significant time effect was present. Subsequently, *post-hoc* analysis was performed using a Bonferroni's multiple comparisons test assuming a significance level of 5%. All data are presented as means ± standard error.

## Results

### Clinical Signs

All OF heifers showed ruminal fluid pH below 5.0 from 12 to 36 h (unpublished data) and distinct symptoms of acute ruminal and systemic acidosis, including depression, loss of appetence, watery diarrhea, and transient fever, which was coincide with previously described (1, 2). These clinical signs were first observed between 6 h and12 h depending on individual heifers and gradually alleviated between 48 and 72 h.

### Neutrophil Oxidative Burst

**Figure 1:**
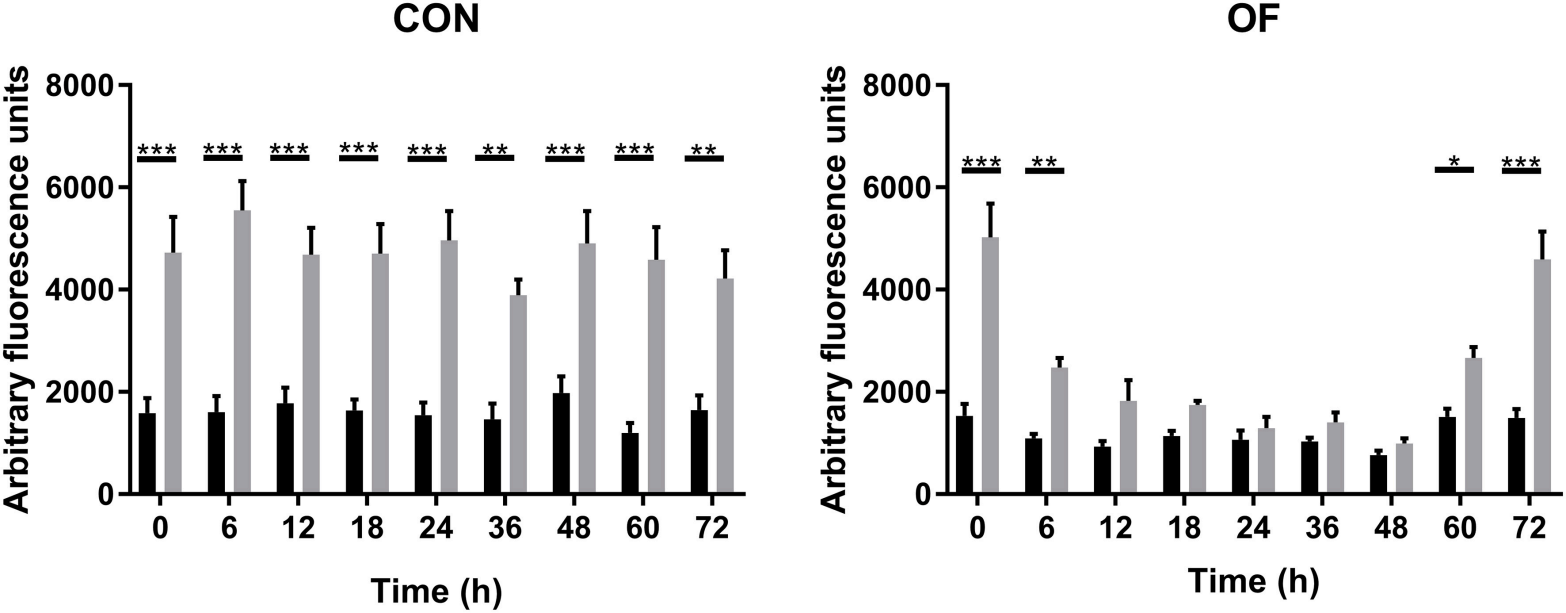
Arbitrary fluorescence of PMN reactive oxygen species (ROS) production over time of heifers with oligofrutose overload (OF, *n* = 6) or tap water (CON, *n* = 6). Supportive therapy was provided in the form of calcium borogluconate at 18 h, and Ringer's solution and sodium bicarbonate at 18 and 24 h after OF overload. ROS production was measured by fluorospectrophotometry using 2, 7-dichlorofluorescin diacetate (DCF-DA). In CON heifers, endogenous and PMA-induced ROS production of PMN did not change over the experimental time. ^*^p < 0.05, ^**^p < 0.01, and ^***^p < 0.001. Black bars represent endogenous ROS production, and gray bars represent PMA-induced ROS production.

In CON heifers, endogenous and PMA-induced ROS production of PMN did not change over the experimental time. Although OF overload did not affect the endogenous ROS production, a significant decrease in PMA-induced ROS production was observed from 12 to 48 h following OF overload compare CON heifers (Figure 1).

### Inflammation Gene Expression

**Figure 2:**
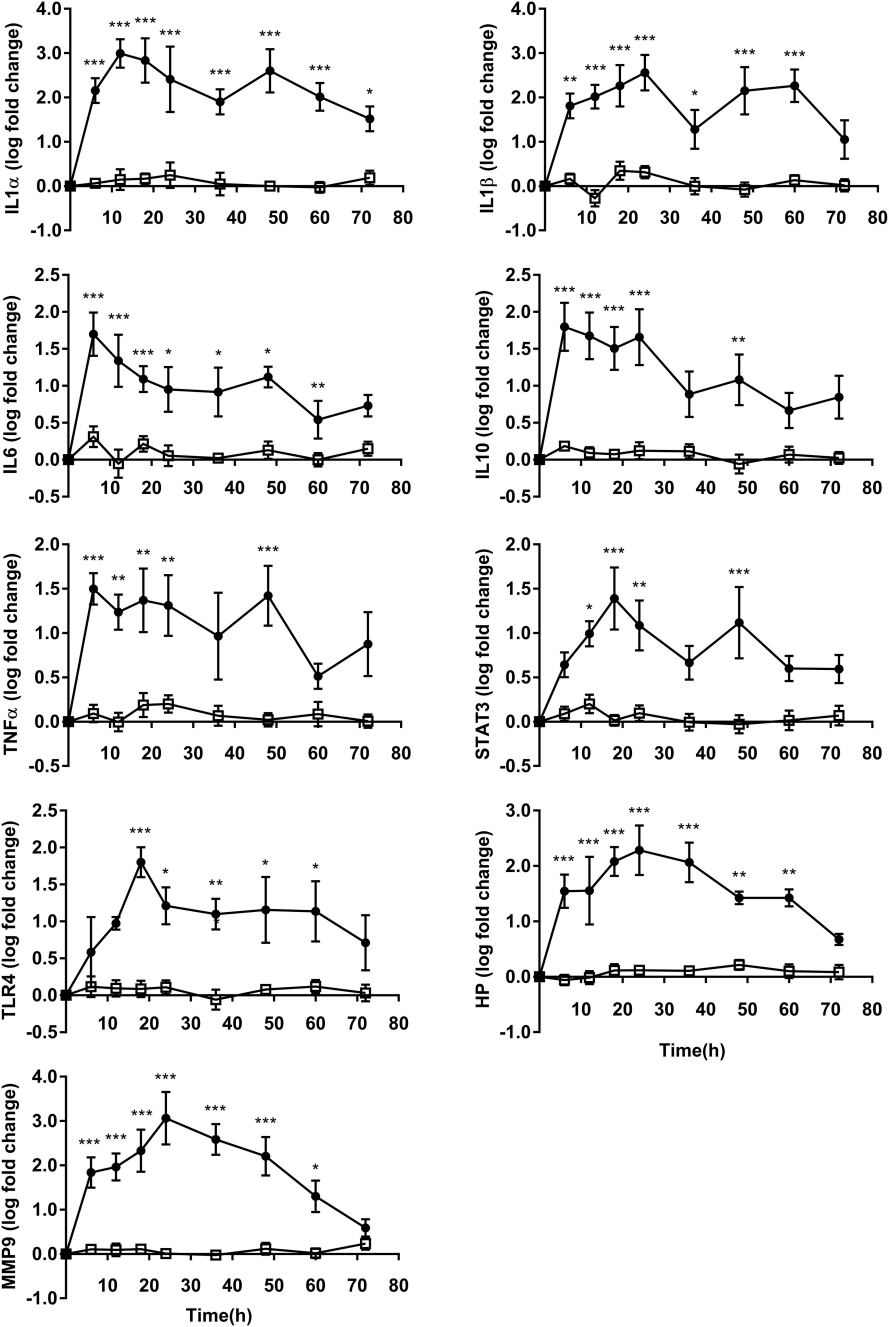
PMN inflammation relative gene expression (log fold-change relative to 0 h) over time of heifers with oligofrutose overload (OF, *n* = 6) or tap water (CON, *n* = 6). Supportive therapy was provided in the form of calcium borogluconate at 18 h, and Ringer's solution and sodium bicarbonate at 18 and 24 h after OF overload. Data were converted to log-2 values prior to statistical analysis. The expressions of all PMN inflammation gene were upregulated after OF overload, while no differences were observed in CON heifers. *P*-value indicate significant time effects within groups. ^*^p < 0.05, ^**^p < 0.01, ^***^p < 0.001, □ = CON; ∙ = OF.

The expressions of all PMN inflammation gene were upregulated after OF overload, while no differences were observed in CON heifers. In OF heifers, gene expression for IL6, IL10, and TNFα peaked at 6 h, and gene expression for IL1α peaked at 12 h; then gene expression for TLR4 and STAT3 peaked at 18 h, followed by IL1β, HP, and MMP9 at 24 h. No changes were found at 60 and/or 72 h in the expression of IL1β, IL6, IL10, TNFα, STAT3, TLR4, MMP9, and HP. IL1α (*P* < 0.05) still showed an increase after OF overload at 72 h compared to CON heifers (Figure 2).

### Adhesion and Migration Gene Expression

**Figure 3:**
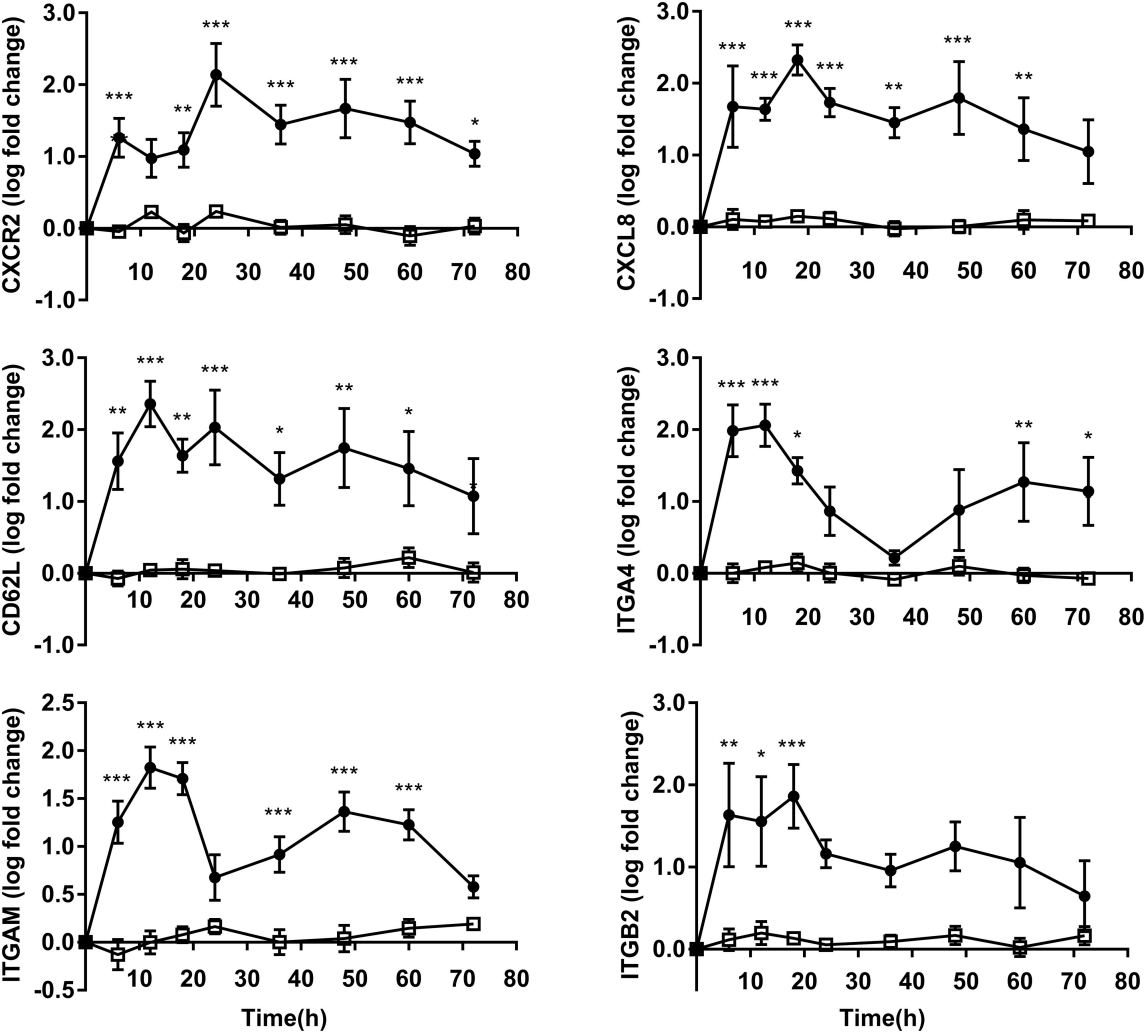
PMN adhesion and migration relative gene expression (log fold-change relative to 0 h) over time of heifers with oligofrutose overload (OF, *n* = 6) or tap water (CON, *n* = 6). Supportive therapy was provided in the form of calcium borogluconate at 18 h, and Ringer's solution and sodium bicarbonate at 18 and 24 h after OF overload. Data were converted to log-2 values prior to statistical analysis. *P*-value indicate significant time effects within groups. ^*^p < 0.05, ^**^p < 0.01, ^***^p < 0.001, □ = CON; ∙ = OF.

The obtained results showed upregulation in OF heifers compared to controls among genes linked to both adhesion and migration. The expressions of CD62L, ITGA4, and ITGAM attained maximum values at 12 h after OF overload. Subsequently, the expressions of CXCL8 and ITGB2 attained maxima at 18 h, and then the CXCR2 expression peaked at 36 h in OF heifers. The expressions of CXCR2 (*P* < 0.05) and ITGA4 (*P* < 0.05) were still elevated above that of the controls at 72 h after OF overload (Figure 3).

### Oxidative Stress and Eicosanoids Gene Expression

**Figure 4:**
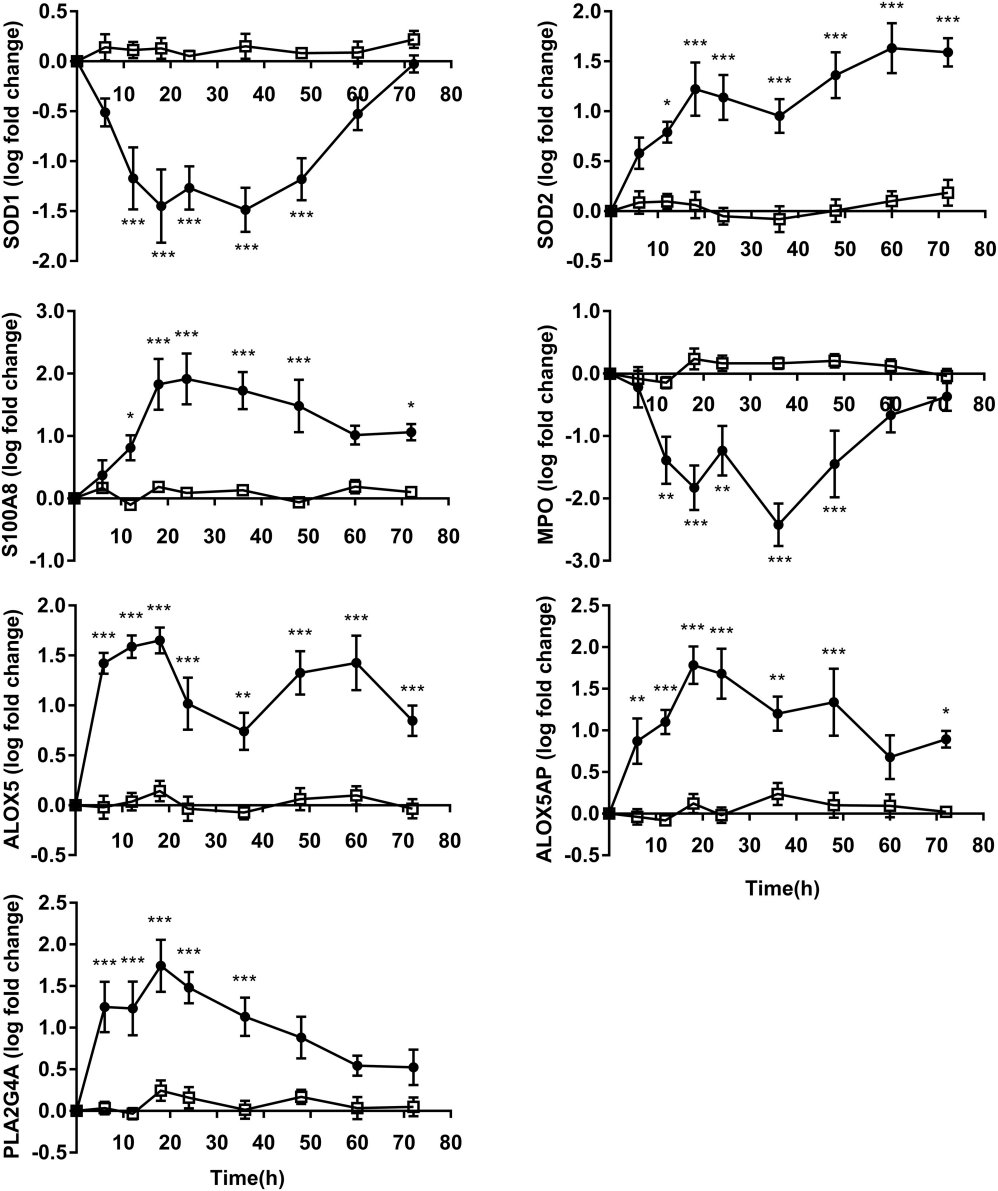
PMN oxidative stress and eicosanoids gene expression (log fold-change relative to 0 h) over time of heifers with oligofrutose overload (OF, *n* = 6) or tap water (CON, *n* = 6). Supportive therapy was provided in the form of calcium borogluconate at 18 h, and Ringer's solution and sodium bicarbonate at 18 and 24 h after OF overload. Data were converted to log-2 values prior to statistical analysis. P value indicate significant time effects within groups. ^*^p < 0.05, ^**^p < 0.01, ^***^p < 0.001, □ = CON; ∙ = OF.

The expressions of SOD1 and MPO indicated that these decreased from time points 18 h to 36 h before returning to levels similar to 0 h. The expression of SOD2 continued to elevate from 12 to 72 h compare to CON. The expressions of S100A8 showed increase at 12 h and attained maximum values at 24 h after OF overload. S100A8 (*P* < 0.05) expression was still increased in OF heifers compared to CON at 72 h, while no difference was observed in MPO expression between groups occurred at 72 h and SOD1 expression from 60 to 72 h (Figure 4).

Gene expression for ALOX5, ALOX5AP, and PLA2G4A increased at 6 h and peaked at 18 h. The expression of ALOX5 remained higher level but decreased during 18–36 h, and then showed increase from 36 to 72 h compared to CON. ALOX5AP (*P* < 0.05) also showed increase at 72 h compared to CON. The differences of PLA2G4A expression levels was not significant from 48 to 72 h between OF and CON (Figure 4).

### Metabolism and Receptor Gene Expression

**Figure 5:**
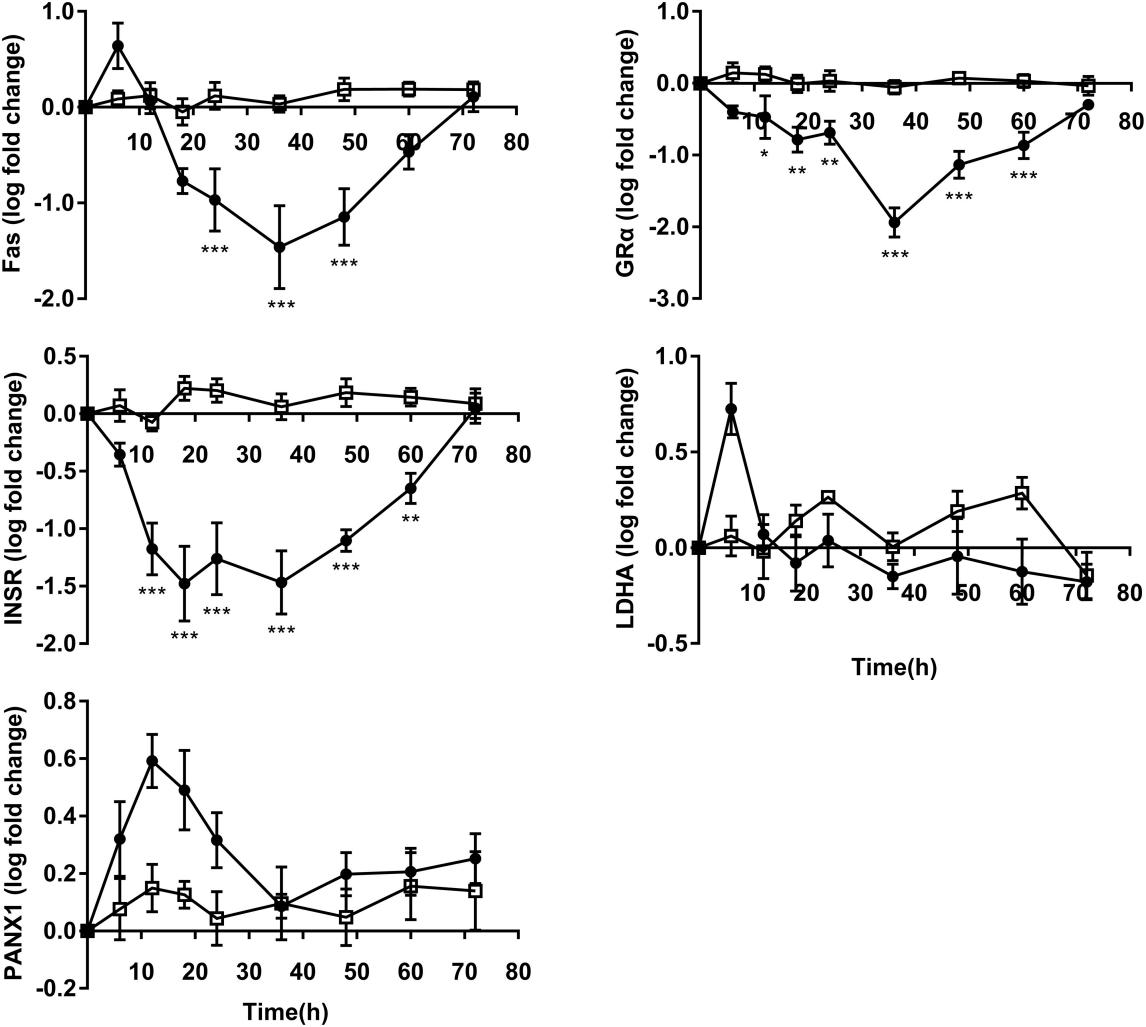
PMN metabolism and receptor relative gene expression (log fold-change relative to 0 h) over time of heifers with oligofrutose overload (OF, *n* = 6) or tap water (CON, *n* = 6). Supportive therapy was provided in the form of calcium borogluconate at 18 h, and Ringer's solution and sodium bicarbonate at 18 and 24 h after OF overload. Data were converted to log-2 values prior to statistical analysis. *P*-value indicate significant time effects within groups. ^*^p < 0.05, ^**^p < 0.01, ^***^p < 0.001, □ = CON; ∙ = OF.

GRα and INSR expressions decreased significantly from 12 to 60 h following OF administration compared to controls. The expression of Fas was downregulated from 24 to 48 h in OF heifers compared to controls. No differences were observed in the expression of LDHA and PANX1 between groups (Figure 5).

## Discussion

To date, the change of bovine PMN immune gene expression has not been studied in a model of OF overload. In the presented experiment, changes of ROS production and genes expression were investigated during the course of OF overload induced ARA. Although gene expression does not necessary reflect the expression of these proteins, the aim was to gather further insight into the role of PMN involved in bovine ruminal acidosis. In addition, considering animal welfare, the experimental design was similar to previous studies that developed supportive therapy to overcorrect severe acidosis at 18 and 24 h after OF overload (1). While the supportive therapy that was utilized in the current study may affect PMN development, this does not affect the overall conclusion of this study.

In this experiment, the expressions of inflammatory cytokines IL1α, IL1β, IL6, and IL10 were increased in OF heifers compared with CON heifers from 6 h up to 72 h after challenge in some cases, which was consistent with a model for OF overload in horses which 10 g/kg amount of OF was given from 8 to 24 h (15). These cytokines play an important role in the innate immune response; however, plasma cytokine concentrations are rarely measured for the cattle with ARA. The plasma concentrations of IL1β, IL6, and TNFα were elevated under the SARA condition induced by reduced forage to concentrate ratio in 4 weeks (7). IL1α and IL1β are able to respond during the initiation of sterile inflammation (16). In horses, the plasma IL1β level increased at 2 h post-prandial feeding with a high starch and sugar diet (17). IL6 plays a complex role related to acute septic shock. A report showed increased levels of IL1β and IL6 in the synovial fluid at 9 and 24 h after OF (13 g/kg) overload in heifers, but not in the plasma (18). In the present study, higher doses of alimentary OF were offered at 17 g/kg of BW; thus, the intensity of the inflammatory response needs further research. IL10 plays a central role in the prevention of excessive tissue damage. Upregulation of both IL1β and IL10 in PMN were found in cows fed with high energy diets, which has been speculated to be caused by the higher concentrations of NEFA and BHBA in the blood (8). Similarly, the plasma NEFA concentration that was not measured in this study also increased in steers and sheep with acute acidosis, which could have an effect on PMN function (19, 20).

Consistent with reports in cows fed higher energy diets, a higher expression of STAT3 was detected in PMN (8). The STAT3 pathway has been shown to orchestrate the inflammatory response through cross-talk with pattern-recognition receptor pathways that recognize pathogen-associated molecular patterns (21). TLR4 is important for the recognition of Gram-negative bacteria. In clinically SARA-positive cows, TLR4 had a higher relative mRNA level (22). Although plasma LPS was not measured in the current study, the upregulated TLR4 expression indicates the possibility that LPS was involved in the inflammatory response of OF-induced ARA. The presence of LPS in the blood has been shown in experimentally induced SARA (5–7). It has also been shown that plasma LPS was increased 8 h after OF overload and declined after 12 h in horses (23).

Haptoglobin (HP) has been a frequently investigated in acute phase reaction proteins in cattle systemic disease. Upregulated HP coincided with the results of plasma concentrations during OF overload (17 g/kg BW) in dairy cattle (24). HP was also markedly increased in SARA cows (7). In general, MMP9 was primarily expressed by inflammatory cells (particularly neutrophil) and is used to degrade the extracellular matrix at the inflammatory site. *In vitro* experiments showed that 2 mM D-lactic acid induced the release of MMP9 (25). The activity of MMP9 in the synovial fluid was also dramatically increased 24 h post-OF overload in heifers, which could be attributed to PMN migration (3).

Directional migration of PMN in response to a chemoattractant gradient was essential for the delivery of PMN to an inflammatory site (26). The current experiment investigated the neutrophil chemoattractant CXCL8 (IL8) and its cell-surface G-protein-coupled chemokine receptor, CXCR2. In this current study, the upregulated expressions of CXCL8 and CXCR2 indicates that PMN had stronger chemotaxis. This can cause PMN rapidly recruited to inflamed tissues, such as an influx of PMN into synovial fluid after OF overload in heifers (18). CXCL8 not only played a role in chemotaxis, but also in the release of alkaline phosphatase from secondary granules and the production of ROS by PMN (26). During the PMN early recruitment process, CD62L (L selectin) and integrin were responsible for the attachment of PMN to the endothelium. In the present study, higher expression levels of CD62L, ITGA4 (CD49d), ITGAM (CD11b), and ITGB2 (CD18) were observed following OF overload. This agreed with a previous study, which observed a reduction in PAF-induced L selectin shedding during OF-induced ARA (2). This indicates that the transendothelial migration of PMN plays an important role in the development of ARA. Interestingly, these adhesion receptors (ITGA4, ITGAM, and ITGB2) seemed to be influenced by supportive therapy and declined at 18 h. This could be due to the mild relief of lactic acidosis, as indicated by the upregulated CD11b expression of bovine neutrophil *in vitro* experiments (9, 25).

While PMN ROS generation was critical to their role in host defense, ROS also contributed to the deleterious injuries associated with the inflammation elicited in response to bacterial infections (27). The result of ROS production agreed with a published ARA experiment after OF (13 g/kg) overload in heifers (2). ROS production remarkably decreased from 12 to 48 h after OF overload. This suggests that the superoxide anion synthesis of PMN was inhibited after OF overload, while it contradicts the upregulated expression of S100A8. S100A8/9 is known to regulate NADPH oxidase activity, which is the major source of ROS in neutrophil. Similar changes also occurred during the periparturient period that ROS production by PMN stimulated with PMA decreased prominently and reached the lowest level at calving (28, 29), although the expression of S100A8/9 had increased compared to the prepartum (8, 30); thus, we postulate that the decrease in PMA-induced ROS production was likely caused by OF overload, which results in increased production of ROS *in vivo*, yet exhausted PMN was unable to produce more additional ROS in response to the PMA stimulation. SOD can catalyze the dismutation of superoxide into hydrogen peroxide, which is reduced to water by catalase (27). The three isoforms of SOD (SOD1, SOD2, and SOD3) reside in the cytoplasm, mitochondria, and extracellular, respectively (31). This study showed a persistent increase in SOD2 expression but a decreased expression of SOD1. This contrasting change was also reported in other studies about bovine neutrophil (32, 33). It was difficult to explain the downregulated SOD1 due to its unclear role. It has been suggested that increased SOD1 activity elevates H_2_O_2_ levels which becomes toxic (31). Higher expressions of SOD2 suggests more pronounced state of oxidative stress after OF overload, which was also found in the PMN of cows on high energy diets (8). The active MPO released during neutrophil degranulation was capable to generate ROS and local MPO activity may reduce nitric oxide synthase activity, thus leading to microvascular dysfunction. Surprisingly, the expression of MPO was downregulated in OF heifers from 12 to 48 h. It has been reported that plasma MPO activity and neutrophil MPO mRNA expression decreased over the early post-partum period (34). Recently, it has been reported that neutrophil transmigration and adhesive function were enhanced and tissue damage was attenuated in MPO knockout mice under sterile inflammatory conditions (35). Therefore, downregulated MPO may contribute to a prolonged inflammatory reaction. More research is needed to investigate the role of oxidative stress in ARA.

Eicosanoids, including prostaglandins and leukotriene synthesis, are metabolized from arachidonic acid. The upregulation of PLA2G4A, ALOX5, and ALOX5AP in PMN suggested that the availability of arachidonic acid increased during OF-induced ARA. The level of blood arachidonic acid was elevated in experimentally induced ARA (36). Furthermore, prostaglandins E_2_ was also increased in the synovial fluid of heifers with ARA (18). In addition, PMN showed higher gene expression related to arachidonic acid (ALOX5AP and PLA2G4A) in cows fed with high energy diets (8).

The downregulation of Fas and GRα could be attributed to the plasma cortisol level, which had increased at 6 h and remained elevated for up to 48 h during OF overload in heifers, indicating that OF overload induces stress or pain (37). Blood cortisol during stress is associated with an increase in circulating neutrophils and nearly five-fold reduction in Fas expression (38, 39). It has also been shown that GRα activation by glucocorticoids in circulating neutrophils rapidly decreases GRα expression (40). Similarly, the plasma cortisol levels were also increased in cows and goats with SARA (41). In addition, an *in vitro* experiment showed that extracellular acidification delayed human neutrophil apoptosis (42). These results suggest that the increased lifespan of PMN provides more time to implement their anti-inflammatory function and cortisol plays an important role in the pathogenesis of bovine ARA. Unexpectedly, the expression of INSR decreased from 12 to 60 h. Although an *in vitro* experiment suggested that insulin had no effect on neutrophil function in dairy cows (43), the relationship between INSR mRNA expression and blood insulin concentration remains unclear. Although systematic insulin and glucose concentration were not different, severe hyperglycemia and hypoinsulinemia were found in steers with acute acidosis before euthanization (19). Long-term metabolic changes during bovine SARA are complex, and requires further research.

## Conclusion

This study of PMN gene expression provides new information on OF-induced bovine ARA. The results indicate that PMN genes that are involved in inflammation, migration, migration, adhesion, oxidative stress, eicosanoids, metabolism, and receptor binding were altered during OF overload. Further research should investigate PMN-related protein abundance in plasma during OF overload.

## Data Availability Statement

The raw data supporting the conclusions of this article will be made available by the authors, without undue reservation, to any qualified researcher.

## Ethics Statement

This animal study was reviewed and approved by Institutional Animal Care and Use Committee of Northeast Agricultural University.

## Author Contributions

SL, JD, LJ, MH, QS, YL, and XZ performed experiment and analyzed data. JZ, JD, and SL conceived the animal experiments. SL wrote the manuscript. All authors approved the final version of the manuscript.

## Conflict of Interest

The authors declare that the research was conducted in the absence of any commercial or financial relationships that could be construed as a potential conflict of interest.
