# Supplementary material for: Dynamic ROS Production and Gene Expression of Heifers Blood Neutrophil in a Oligofructose Overload Model
Source: Front Vet Sci. 2020 Apr 21;7:211. doi: 10.3389/fvets.2020.00211 (PMC7186304; doi:10.3389/fvets.2020.00211)
Supplement: Supplementary file 1 [file Table_1.DOCX]

**Supplemental Table:** Gene symbol, Sequence, Amplicon size, and National Center for Biotechnology Information (NCBI) accession number of primers used to analyze gene expression by quantitative PCR

| **Gene symbol** | **Sequence 5` → 3`**  **Forward (F) or reverse (R)** | **Amplicon size (bp)** | **NCBI accession no.** | **References** |
| --- | --- | --- | --- | --- |
| *ACTB* | F: ACTTGCGCAGAAAACGAGAT  R: CACCTTCACCGTTCCAGTTT | 123 | BT030480 | ([1](#_ENREF_1)) |
| *ALOX5* | F: TCATCAACCGCTTCATGCA  R: GTGATTCATGACCCGCTCAGA | 127 | NM_001192792.2 | ([2](#_ENREF_2)) |
| *ALOX5AP* | F: ACAAGGTGGAGCACGAAAGC  R: ACACAGTTCTGGTTGGCAGTGT | 100 | NM_001076293.2 | ([2](#_ENREF_2)) |
| *CD62L* | F: CCGATTGCTGGACTTACCAT  R: CCAAGTCCACACCCCTTCTA | 194 | NM_174182 | ([1](#_ENREF_1)) |
| *CXCL8* | F: GACAGCAGAGCTCACAAGCATCT  R: AAGCTGCCAAGAGAGCAACAG | 105 | NM_173925.2 | ([2](#_ENREF_2)) |
| *CXCR2* | F: GGCACTGGGTCAAGTTCATATGT  R: CGGAGTACGGTGGTTGATAGG | 102 | NM_174360.2 | ([3](#_ENREF_3)) |
| *Fas* | F: AGTTGGGGAGATGAATGCTG  R: CCTGTGGATAGGCATGTGTG | 171 | NM_174662 | ([1](#_ENREF_1)) |
| *GAPDH* | F: GGGTCATCATCTCTGCACCT  R: GGTCATAAGTCCCTCCACGA | 176 | DQ402990 | ([1](#_ENREF_1)) |
| *GRα* | F: CCATTTCTGTTCACGGTGTG  R: CTGAACCGACAGGAATTGGT | 132 | AY238475 | ([1](#_ENREF_1)) |
| *HP* | F: TGGTCTCCCAGCATAACCTC  R: AGGGTGGAGAACCACCTTCT | 185 | BC109668 | ([1](#_ENREF_1)) |
| *IL10* | F: ACAGGCTGAGAACCACGGGC  R: GACACCCCTCTCTTGGAGCTCACT | 175 | NM_174088.1 | ([1](#_ENREF_1)) |
| *IL1α* | F: TCATCCACCAGGAATGCATC  R: AGCCATGCTTTTCCCAGAAG | 300 | NM_174092 | ([4](#_ENREF_4)) |
| *IL1β* | F: ATTCTCTCCAGCCAACCTTCATT  R: TTCTCGTCACTGTAGTAAGCCATCA | 100 | NM_174093.1 | ([2](#_ENREF_2)) |
| *IL6* | F: ATGACTTCTGCTTTCCCTACCC  R: GCTGCTTTCACACTCATCATTC | 180 | NM_173923 | ([5](#_ENREF_5)) |
| *INSR* | F: CCCTTCGAGAAAGTGGTGAACA  R: AGCCTGAAGCTCGATGCGATAG | 84 | XM_005208817.2 | ([6](#_ENREF_6)) |
| *ITGA4* | F: ACAGACAGCTCTGGCATAGTGAGA  R: AGCTGGCATCCAGAAGAAAGC | 102 | NM_174748.1 | ([7](#_ENREF_7)) |
| *ITGAM* | F: GGCTTGTCTCTTGCATTTGCT  R: CCATTTGCATAGGTGTTCTCCTT | 95 | NM_001039957.1 | ([2](#_ENREF_2)) |
| *ITGB2* | F: GACACCCTGAAAGTCACCTACGA  R: GAAGGTGATCGGGACGTTGAT | 108 | NM_175781.1 | ([2](#_ENREF_2)) |
| *LDHA* | F: CAAGAGGTACCACTGCCCAT  R: CACCTTGGCTAAAGGAACCA | 100 | NM_174099.2 | ([2](#_ENREF_2)) |
| *MMP9* | F: CCCGACCCGAGTCGATGCAA  R: GCGGCCACAAGGAACAGGCT | 213 | NM_174744.2 | ([1](#_ENREF_1)) |
| *MPO* | F: AGCCATGGTCCAGATCATCAC  R: ACCGAGTCGTTGTAGGAGCAGTA | 105 | NM_001113298.2 | ([2](#_ENREF_2)) |
| *PANX1* | F: AGAAAGCCTCCAGACATTTAATCG  R: AACTCATCCGAGAGGGAGGAA | 124 | NM_001245925.1 | ([2](#_ENREF_2)) |
| *PLA2G4A* | F: CTCCATGTCAAACCCGATGTC  R: GTCAGGCGCCATAAAAGTACCA | 105 | NM_001075864.1 | ([2](#_ENREF_2)) |
| *S100A8* | F: ACACCATGCTGACGGATCTG  R: TCCCTATAGACGGCGTGGTAA | 100 | NM_001113725.2 | ([2](#_ENREF_2)) |
| *SOD1* | F: GGCTGTACCAGTGCAGGTCC  R: GCTGTCACATTGCCCAGGT | 100 | XM_005201085.1 | ([8](#_ENREF_8)) |
| *SOD2* | F: TGTGGGAGCATGCTTATTACCTT  R: TGCAGTTACATTCTCCCAGTTGA | 95 | NM_201527.2 | ([2](#_ENREF_2)) |
| *STAT3* | F: GGTAGCATGTGGGATGGTCTCT  R: GCATCCCTAGAAACTCTGGTCAA | 110 | NM_001012671.2 | ([2](#_ENREF_2)) |
| *TLR4* | F: TGGTAAACCCCAGAGTCCAG  R: GCACAATGCTTGGTACATGG | 164 | NM_174198 | ([9](#_ENREF_9)) |
| *TNFα* | F: CCAGAGGGAAGAGCAGTCCC  R: TCGGCTACAACGTGGGCTAC | 114 | NM_173966.3 | ([2](#_ENREF_2)) |
| *UXT* | F: TGTGGCCCTTGGATATGGTT  R: GGTTGTCGCTGAGCTCTGTG | 101 | BC108205.1 | ([8](#_ENREF_8)) |

**REFERENCES**

1. O'Driscoll K, McCabe M, Earley B: Differences in leukocyte profile, gene expression, and metabolite status of dairy cows with or without sole ulcers**.** *J Dairy Sci* (2015) 98**:**1685-1695. doi: 10.3168/jds.2014-8199

2. Batistel F, Osorio JS, Tariq MR, Li C, Caputo J, Socha MT, Loor JJ: Peripheral leukocyte and endometrium molecular biomarkers of inflammation and oxidative stress are altered in peripartal dairy cows supplemented with Zn, Mn, and Cu from amino acid complexes and Co from Co glucoheptonate**.** *J Anim Sci Biotechnol* (2017) 8**:**33. doi: 10.1186/s40104-017-0163-7

3. Seo J, Osorio JS, Loor JJ: Purinergic signaling gene network expression in bovine polymorphonuclear neutrophils during the peripartal period**.** *J Dairy Sci* (2013) 96**:**7675-7683. doi: 10.3168/jds.2013-6952

4. Gabler C, Drillich M, Fischer C, Holder C, Heuwieser W, Einspanier R: Endometrial expression of selected transcripts involved in prostaglandin synthesis in cows with endometritis**.** *THERIOGENOLOGY* (2009) 71**:**993-1004. doi: 10.1016/j.theriogenology.2008.11.009

5. Herath S, Lilly S, Santos N, Gilbert R, Goetze L, Bryant C, White J, Cronin J, Sheldon I: Expression of genes associated with immunity in the endometrium of cattle with disparate postpartum uterine disease and fertility**.** *Reprod Biol Endocrin* (2009) 7. doi: 10.1186/1477-7827-7-55

6. Gao ST, Guo J, Quan SY, Nan XM, Bu DP: Heat-Stress Decreases Milk Protein through Induction of Mammary Cells Apoptosis of Cows**.** *Chinese J Anim Nutr* (2016) 28**:**1615-1625. doi: 1006-267X(2016)28:5<1615:RYJTGY>2.0.TX;2-Z

7. Moyes KM, Graugnard DE, Khan MJ, Mukesh M, Loor JJ: Postpartal immunometabolic gene network expression and function in blood neutrophils are altered in response to prepartal energy intake and postpartal intramammary inflammatory challenge**.** *J Dairy Sci* (2014) 97**:**2165-2177. doi: 10.3168/jds.2013-7433

8. Abdelmegeid M, Vailati-Riboni M, Alharthi A, Batistel F, Loor J: Supplemental methionine, choline, or taurine alter in vitro gene network expression of polymorphonuclear leukocytes from neonatal Holstein calves**.** *J Dairy Sci* (2017) 100**:**3155-3165. doi: 10.3168/jds.2016-12025

9. O'Loughlin A, McGee M, Waters S, Doyle S, Earley B: Examination of the bovine leukocyte environment using immunogenetic biomarkers to assess immunocompetence following exposure to weaning stress**.** *BMC Vet Res* (2011) 7. doi: 10.1186/1746-6148-7-45
